# Chronic constriction injury-induced changes in circular RNA expression profiling of the dorsal root ganglion in a rat model of neuropathic pain

**DOI:** 10.1186/s12868-022-00745-5

**Published:** 2022-11-15

**Authors:** Wanxia Xiong, Min Wei, Li Zhang, Jie Wang, Fan Liu, Zhiyao Wang

**Affiliations:** 1 Department of Anesthesiology, Zhongshan Hospital, Fudan University, Shanghai, China; 2 Center for Anesthesiology, Beijing Anzhen Hospital, Capital Medical University, Beijing, China; 3 Department of Anesthesiology, Beijing Friendship Hospital, Capital Medical University, Beijing, China; 4 Department of Human Anatomy, Histology and Embryology, Institute of Basic Medical Sciences Chinese Academy of Medical Sciences, School of Basic Medicine Peking Union Medical College, Beijing, China

**Keywords:** Neuropathic pain, Circular RNA, Chronic constriction injury, Dorsal root ganglion, Circular RNA profiling

## Abstract

**Background:**

The pathogenesis of neuropathic pain (NP) has not been fully elucidated. Gene changes in dorsal root ganglia (DRG) may contribute to the development of NP. Circular RNAs (circRNAs) are a class of endogenous noncoding RNAs that form covalently closed loop structures and are crucial for genetic and epigenetic regulation. However, little is known about circRNA changes in DRG neurons after peripheral nerve injury.

**Methods:**

A sciatic nerve chronic constriction injury (CCI) model was established to induce neuropathic pain. We performed genome-wide circRNA analysis of four paired dorsal root ganglion (DRG) samples (L4–L5) from CCI and negative control (NC) rats using next-generation sequencing technology. The differentially expressed circRNAs (DEcircRNAs) were identified by differential expression analysis, and the expression profile of circRNAs was validated by quantitative PCR. Gene Ontology and Kyoto Encyclopedia of Genes and Genomes analyses were performed to predict the function of DEcircRNAs.

**Results:**

A total of 374 DEcircRNAs were identified between CCI and NC rats using circRNA high-throughput sequencing. Among them, 290 were upregulated and 84 were downregulated in the CCI group. The expression levels of nine DEcircRNAs were validated by qPCR. Functional annotation analysis showed that the DEcircRNAs were mainly enriched in pathways and functions, including ‘dopaminergic synapse,’ ‘renin secretion,’ ‘mitogen-activated protein kinase signaling pathway,’ and ‘neurogenesis.’ Competing endogenous RNA analysis showed that the top 50 circRNAs exhibited interactions with four pain-related microRNAs (miRNAs). Circ:chr2:33950934–33955969 was the largest node in the circRNA–miRNA interaction network.

**Conclusions:**

Peripheral nerve injury-induced neuropathic pain led to changes in the comprehensive expression profile of circRNAs in the DRG of rats. DEcircRNAs may advance our understanding of the molecular mechanisms underlying neuropathic pain.

**Supplementary Information:**

The online version contains supplementary material available at 10.1186/s12868-022-00745-5.

## Background

Neuropathic pain develops after injury of the somatosensory system [1, 2]. It is characterized by spontaneous continuous or paroxysmal pain and stimuli-evoked pain [1, 3] and has emerged as one of the most challenging pain syndromes, affecting 7–10% of the population [1]. Moreover, the incidence of neuropathic pain is increasing with the aging of the global population [1]. Despite recent improvements, the management of neuropathic pain remains unsatisfactory, mainly due to an insufficient understanding of the molecular mechanisms underlying the syndrome [2, 4]. Therefore, there is a need to reveal the underlying mechanisms to benefit the clinical management of neuropathic pain.

The dorsal root ganglion (DRG) contains a large proportion of the body’s sensory neurons, which are critical for transducing sensory information from the periphery to the central nervous system [5, 6]. Accumulating evidence has highlighted that the development of neuropathic pain is associated with changes in DRG neurons, such as the release of cytokines and dysregulation of ion channels [5]. In addition to changes in the expression of individual genes, recent studies have highlighted gene expression alterations in DRGs associated with neuropathic pain. After examining gene expression in the DRG of normal rats and peripheral axotomy-operated rats, Xiao et al*.* [7] identified significant differences in the expression of 173 genes, including neuropeptides, ion channels, and synaptic vesicle proteins. Reinhold et al*.* [8] used fluorescent neuronal tracers to confirm the differential expression patterns between damaged DRG neurons and adjacent neurons [7]. Furthermore, universal alterations of microRNA (miRNA) expression in DRG have been demonstrated in neuropathic pain. Li et al*.* [9] identified a total of 114 differentially expressed miRNAs in the DRG of a neuropathic pain rat model [8]. In another study, the expression of 42 miRNAs was significantly altered in the DRG of a streptozotocin-induced diabetic neuropathic pain mouse model [9].

Circular RNAs (circRNAs) are a large family of covalently closed RNA molecules with regulatory roles in gene transcription, miRNA function, and protein production [10–12]. They are expressed in diverse cell types and preferentially expressed in neural tissues [12]. The expression levels of circRNAs are dynamically modulated in neurons where are essential for synaptic plasticity and neuronal function [13]. In a recent study, 469 circRNAs were found to be differentially expressed in the spinal dorsal horn of chronic constriction injury (CCI) rats [14]. Wang et al. found that circHIPK3, which is involved in neuroinflammation, was associated with NP grade in type 2 diabetic patients. Silencing circHIPK3 can alleviate pain in diabetic NP rats [15].

Zhang et al. found that cytoplasmic circAnks1a can act as an endogenous miR-324-3p sponge, resulting in increased VEGFB expression, which was positively correlated with pain behavior induced by nerve injury [16]. In a CCI model, Zhang et al. confirmed that circ_0005075 can target miR-151a-3p, contributing to the pain behavior of rats by inducing NOTCH2 expression [17]. In another study, Cai et al. observed that ciRS-7, sponged to miR-135a-5p, contributed to the development of NP by upregulating autophagy and inflammation [18]. All of these study results indicated that alterations in the expression of circRNAs may play roles in neuropathic pain [14]. However, knowledge about the altered expression of circRNAs in the DRG of rats with neuropathic pain is limited.

To gain a global view of aberrant circRNA expression in the DRG of CCI rats, we established the CCI model and evaluated circRNA expression levels in the DRG using next-generation sequencing technology. Differentially expressed circRNAs (DEcircRNAs) between CCI and NC rats were identified and validated by quantitative PCR (qPCR). Functional annotation was implemented to explore the biological function of DEcircRNAs.

## Materials and methods

### Animals

Adult Sprague Dawley rats (male, 200–250 g) were acquired from the Experimental Animal Center of Fudan University (Shanghai, China). Age-matched rats were blinded and randomly assigned to CCI or negative control (NC) groups (*n* = 7/group). Rats were housed in a pathogen-free condition with a standard 12-h light/dark cycle and free access to rodent food and water. The animal experimental protocols (No. 2019-079) were approved by the Institutional Animal Care and Use Committee of Fudan University and complied with the ethical guidelines of the International Association for the Study of Pain to ensure minimal animal use and discomfort. All rats were acclimatized to laboratory conditions before the tests. All behavioral experiments were carried out between 9:00 a.m. and 12:00 p.m.

### Experimental design

Our research goal was to examine the changes in circRNA expression in the DRG after surgery. All rats were randomized to the NC group and CCI surgery group, and mechanical allodynia and thermal hyperalgesia were assessed after surgery. All animals were euthanized by an overdose of sodium pentobarbital in 14 days after surgery. L4–L5 DRGs of rats ipsilateral to the nerve surgery were harvested for subsequent total RNA extraction.

### Rat model of neuropathic pain

The CCI rat model was produced under sodium pentobarbital anesthesia (40 mg/kg, intraperitoneal injection) based on a procedure established by Bennett and Xie [19]. The sciatic nerves on the left side were exposed, and then ligatures were tied loosely around the nerve with approximately 1 mm space between knots. Rats in the NC group were subjected to sciatic nerve exposure without ligation.

## Behavioral test

### Paw withdrawal mechanical threshold (PWMT) test

According to previous studies [2, 20, 21], PWMT in response to mechanical stimuli was carried out with an electronic von Frey paw pressure anesthesiometer (IITC Life Science, Woodland Hills, CA, USA), which consists of a hand-held force transducer with a fixed 200 μm diameter tungsten wire tip. The experimenter applied the electronic von Frey probe tip perpendicularly to the plantar surface of the hind paw as described previously. Brisk withdrawal or flinching evoked by applications was considered a positive response. The test was performed 1 day before and 1, 3, 5, 7, 9, and 14 days after surgery. The three measurements of the mechanical threshold were averaged.

### Paw withdrawal thermal latency (PWTL) tests

According to the protocol described previously [2, 20, 21], PWTL in response to radiant heat was carried out using a heat-mediated pain stimulator (Model 336; IITC Life Science). The time from onset of application to hind paw withdrawal was measured, and each rat was tested three times in 10-min intervals. The three measurements of thermal latency were averaged.

The test was also performed 1 day before and 1, 3, 5, 7, 9, and 14 days after surgery.

### RNA isolation and quality control

The rats were anesthetized with sodium pentobarbital (40 mg/kg, intraperitoneal injection) and sacrificed by cervical dislocation at the end of the behavioral test. L4–L5 DRGs were excised from a single rat in each group (CCI and NC) and rapidly frozen at − 80 °C. Total RNA of each DRG sample was isolated using TRIzol reagent (Invitrogen, Carlsbad, CA, USA) following the manufacturer’s instructions. The quality and quantity of total RNA samples were measured with an ND-1000 NanoDrop spectrophotometer (Nano-Drop Technologies, Wilmington, DE, USA). The integrity of each RNA sample was assessed by denaturing agarose gel electrophoresis.

### Next-generation RNA sequencing analysis

Total RNA from four matched DRG samples of CCI and NC rats was treated with the Epicenter Ribo-Zero rRNA Removal Kit (Illumina, San Diego, CA, USA) and RNAse R (Epicenter, Madison, WI, USA) to remove ribosomal and linear RNA, respectively. A strand-specific sequencing library was prepared by using the NEBNext Ultra Directional RNA Library Prep Kit (Illumina). Then, RNA sequencing (RNA-seq) was performed on the Illumina HiSeq 2500 system following the manufacturer’s recommendations. All sequencing procedures and analyses were performed by RiboBio (Guangzhou, China). High-throughput sequencing (HTSeq) software was used to count the numbers of sequence reads mapped to each circRNA [22]. TopHat mapping and TopHat-Fusion mapping reads were used by RPM (Reads Per Million mapped reads) to calculate the expression of individual circRNA [23, 24]. The total number of reads spanning back-spliced junctions was used as an absolute measure of circRNA abundance. ANNOVAR was used to analyse the functional elements from which the circRNAs were derived [25]. DEcircRNAs between the CCI and NC groups were identified using fold change (FC) filtering and Q value. The Q value was acquired by adjusting the p value using the Benjamini‒Hochberg method. We defined the statistical criteria for selecting aberrantly expressed circRNAs using a q-value of < 0.001 with an FC of ≥ 2 or ≤ 0.5. The cluster profile of DEcircRNAs was revealed by two-way hierarchical clustering analysis based on their expression values.

### Functional annotation

The possible functions of circRNAs were assumed to be related to their host genes. Kyoto Encyclopedia of Genes and Genomes (KEGG) [26–28] and Gene Ontology (GO) [29] analyses were performed for DEcircRNAs to reveal neuropathic pain-associated biological pathways and functions. Fisher’s exact test was used to compute the statistical significance of the enrichment. The selection criterion for significantly enriched terms was set at FDR < 0.05.

### qPCR

cDNA samples were prepared by reverse transcribing total RNAs using the PrimeScript RT Reagent Kit (TaKaRa, Tokyo, Japan). DEcircRNAs that were expressed in all samples and showed significant expression differences were selected for qPCR analysis. The expression levels of the selected DEcircRNAs were validated by qPCR assays using SYBR Green qPCR Master Mix (ABI, Los. Angeles, CA, USA) and specific primer sequences (Additional file 1: Table S1). The expression levels of circRNAs were normalized to GAPDH expression and analysed by the 2^−ΔΔCt^ method.

### Statistical analyses

Values were expressed as group mean and standard errors (mean ± SEM) and analysed using GraphPad Prism 8 (GraphPad Software, San Diego, CA, USA), Microsoft Excel (Microsoft, Washington, DC, USA), and R software version 3.5.3 (http://www.r-project.org/). Q-value of < 0.001 with an FC of ≥ 2 or ≤ 0.5 was used to identify significant differences in circRNA expression. According to previous studies [30, 31], two-way ANOVA followed by Bonferroni’s post-hoc tests was used to analyse the behavioral data. *p* < 0.05 was considered statistically significant, *p < 0.05, **p < 0.01, compared with NC groups.

## Results

### Development of mechanical and thermal hypersensitivity after CCI

**Fig. 1:**
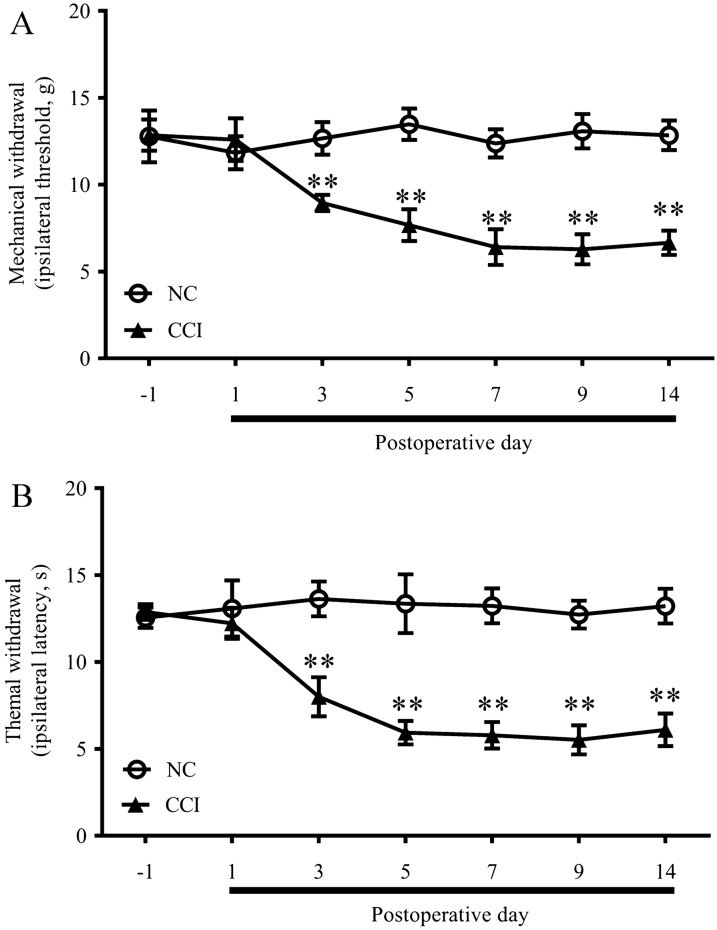
Chronic constriction injury-induced nociceptive behavior of rats. Mechanical allodynia (**a**) and thermal hyperalgesia (**b**) at each time point after CCI surgery. *n* = 7/group. All data are represented as the mean ± SEM, two-way ANOVA tests followed by Bonferroni’s post-hoc tests, **p < 0.01, CCI versus negative control (NC)

To reveal circRNA expression changes induced by neuropathic pain, rat CCI models were established. Mechanical allodynia and thermal hyperalgesia were used to evaluate the models. As revealed by PWT and PWL tests, PWT and PWL were significantly decreased in the CCI group from postoperative day (POD) 3 to POD14 compared with the NC group (Fig. 1).

### Overview of circRNA profiles

**Fig. 2:**
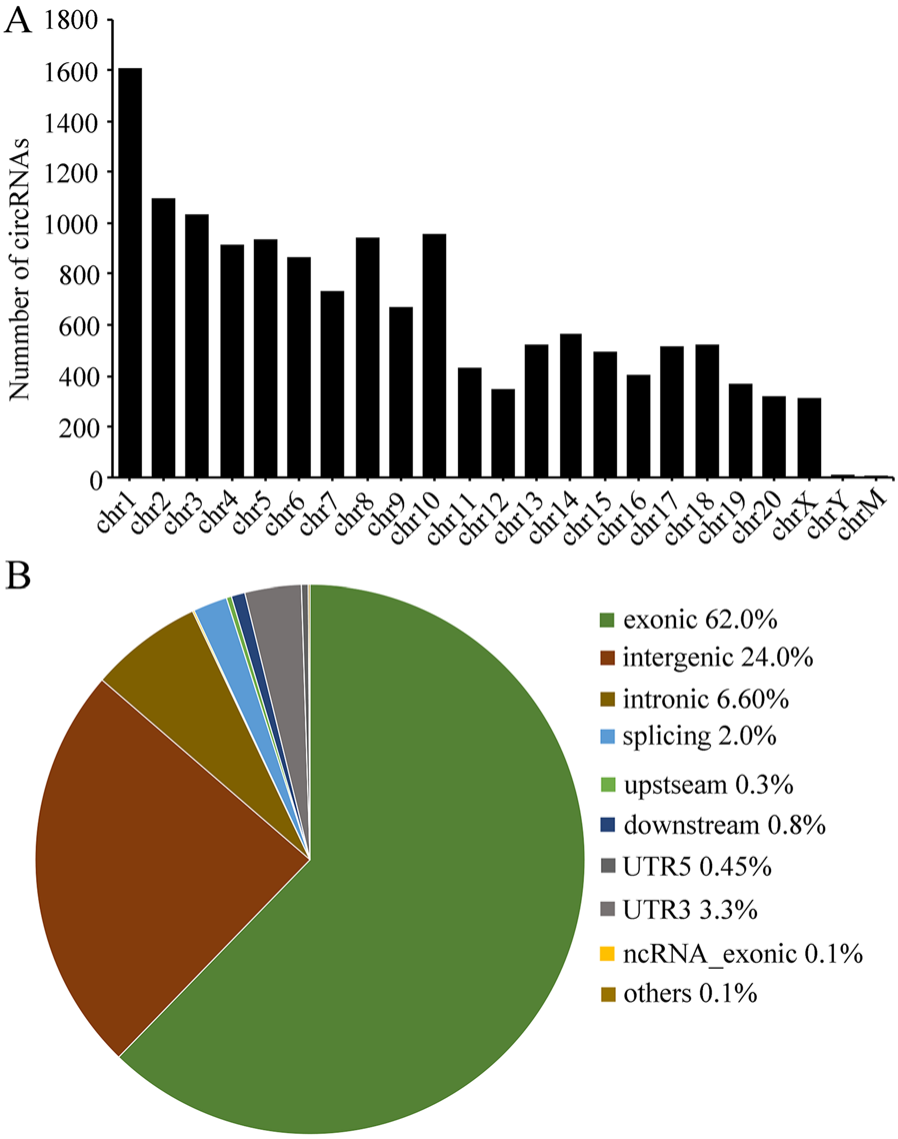
Chromosome distribution of circular RNAs. **a** Chromosome distribution of circular RNAs (circRNAs). **b** Genome distribution of circRNA sequence fragments

RNA-seq was performed to profile circRNA expression in four paired DRG samples. A total of 13,172 circRNAs were detected in the L4–L5 DRG samples from the CCI and NC groups (Additional file 1: Table S2). Among these, 7514 circRNAs contained at least three unique back-spliced reads. According to their host gene location, the analysis of chromosome distribution showed that these 7514 circRNAs were derived from all chromosomes, including the mitochondrial genome. Chromosomes 1 to 3 produced more than 3000 circRNAs, and several hundred circRNAs were distributed on other chromosomes. Only 10 circRNAs were detected on chromosome Y, and 4 circRNAs were detected on the mitochondrial genome (Fig. 2a). According to their location on the genome, the host genes of these circRNAs were derived from exonic regions (62.0%), intergenic regions (24.0%), and intronic regions (6.6%) (Fig. 2b).

### CCI induces genome-wide circRNA differential expression in DRGs

**Fig. 3:**
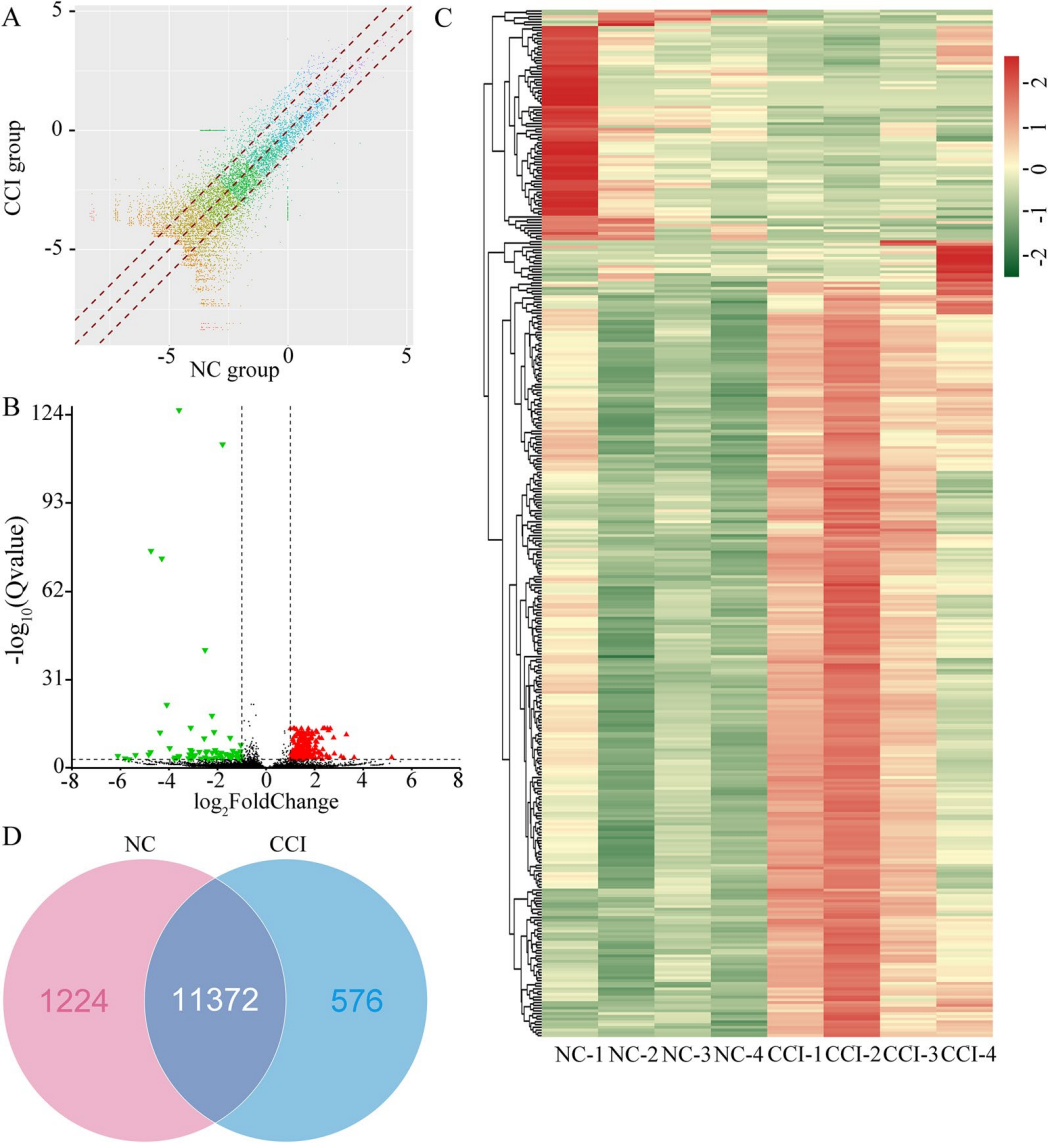
Differentially expressed circRNAs in dorsal root ganglions between the negative control and chronic constriction injury model. **a** Scatter plots showing circular RNA (circRNA) expression differences in the L4–L5 dorsal root ganglions (DRGs) of chronic constriction injury (CCI) rats with respect to the negative control (NC) group. *n* = 4/group. The circRNAs above the top red line or below the bottom red line indicated differential circRNAs (fold change ≥ 2 or ≤ 0.5, and FDR < 0.001) respectively. **b** Volcano plot indicating differentially expressed circRNAs (DEcircRNAs) in the L4–L5 DRGs of CCI models. Red dots represent circRNAs with significant expression, whereas black dots represent circRNAs with no significant difference. **c** Heatmap of DEcircRNAs shows hierarchical clustering of DEcircRNAs of rats in the CCI group compared to the NC group. In the clustering analysis, upregulated and downregulated genes are shown in red and green, respectively. **d** The Venn diagram indicates the number of overlapping and distinct circRNAs between NC and CCI rats

Following differential expression analysis, 374 DEcircRNAs were identified between the CCI and NC groups by using FC filtering (FC ≥ 2 or ≤ 0.5) and FDR (Q < 0.001). Among the DEcircRNAs, 84 were downregulated and 290 were upregulated in the CCI group. A scatter plot was used to show variation in circRNA expression between these two groups of DRG samples (Fig. 3a). Moreover, a volcano plot was drawn to visualize the DEcircRNAs with statistical significance (Fig. 3b). Hierarchical clustering analysis was performed to group samples based on the expression levels of DEcircRNAs across samples. As shown by the heatmap, the CCI and NC samples showed distinguishable circRNA expression profiles. Venn diagram was made according to average of RPM in two groups and indicated the number of overlapping and distinct circRNAs between NC and CCI rats. (Fig. 3c, d, Additional file 2: Table S2 and Additional file 3: Table S3). The top 50 upregulated circRNAs are listed in Table 1, and the downregulated circRNAs are listed in Table 2.

**Table 1:** Top 50 upregulated circRNAs between NC and CCI rats

cicName	Gene symbol	NC	CCI	Log2FoldChange	*p* value	*q* value
rat_circ:chr13:14728984–14734632	*	0.006613447	0.239134343	5.176275102	1.04E−05	0.000197339
rat_circ:chr8:122024123–122051181	*	0.026453788	0.327144217	3.628380535	1.08E−05	0.000204393
rat_circ:chr17:5142807–5148631	*	0.102932557	1.020292962	3.309212177	3.53E−14	1.92E-12
rat_circ:chr3:16676098–16677678	*	0.038958884	0.357541925	3.198088158	1.75E−05	0.000311119
rat_circ:chr10:83601126–83611053	*	0.048021776	0.409332141	3.091511283	5.04E−06	0.000100524
rat_circ:chr4:133073964–133096725	Shq1	0.079302196	0.557523925	2.813600993	4.06E−07	9.77E−06
rat_circ:chr6:12442152–12468485	Gtf2a1l	0.158363447	1.090678555	2.783914689	4.89E−13	2.40E−11
rat_circ:chr2:3165816–3262689	*	0.269462862	1.688134016	2.647271084	2.22E−16	1.41E−14
rat_circ:chr4:127707965–127709016	Suclg2	0.07789907	0.475982505	2.611230542	8.46E−06	0.000162135
rat_circ:chr1:207212800–207228383	Dock1	0.250908366	1.490557854	2.570619891	1.11E−15	6.95E−14
rat_circ:chr12:628708–632694	N4bp2l2	0.331660809	1.909515799	2.525426408	2.22E−16	1.41E−14
rat_circ:chr9:98293254–98296809	*	0.075039684	0.426190004	2.505771094	1.13E−05	0.000211892
rat_circ:chr6:103783216–103876906	*	0.095921747	0.536135335	2.482667387	1.41E−06	3.06E−05
rat_circ:chr9:21359002–21381023	*	0.351886506	1.877364693	2.415526836	2.22E−16	1.41E−14
rat_circ:chr13:42464681–42484505	*	0.461623763	2.428772129	2.395437745	2.22E−16	1.41E−14
rat_circ:chr17:31092766–31111405	*	0.397313897	2.017431411	2.344168464	2.22E−16	1.41E−14
rat_circ:chr18:15167501–15177828	Mapre2	0.163508464	0.825898788	2.33659967	9.67E−09	3.04E−07
rat_circ:chr1:115688753–115690048	*	0.084999051	0.422172726	2.312314733	6.00E−05	0.000921612
rat_circ:chr1:132003515–132012253	*	0.122526404	0.60044185	2.292931859	1.19E−06	2.64E−05
rat_circ:chr10:51399085–51420055	*	0.265527228	1.296441867	2.287625806	8.00E−13	3.83E−11
rat_circ:chr10:51724607–51734014	Myocd	0.158230338	0.739399801	2.224328417	2.55E−07	6.38E−06
rat_circ:chr18:13870389–13923877	Nol4	0.311141127	1.410542522	2.18060915	4.60E−13	2.27E−11
rat_circ:chr15:16089766–16170268	Fhit	0.174858821	0.791604402	2.178589054	1.11E−07	2.95E−06
rat_circ:chr10:32437291–32442282	Sgcd	0.118076637	0.53278429	2.173828	1.13E−05	0.000211892
rat_circ:chr4:129756394–129778825	*	0.173758692	0.772875498	2.153150883	1.75E−07	4.49E−06
rat_circ:chr14:94275506–94356512	*	2.000034951	8.784498579	2.134934728	0	0
rat_circ:chr10:77840596–77845575	*	0.184956873	0.804092283	2.120172172	1.87E−07	4.73E−06
rat_circ:chr3:63039411–63058267	Pde11a	0.396002984	1.710669556	2.110977899	5.55E−15	3.28E−13
rat_circ:chr7:64050812–64065194	Srgap1	0.318355006	1.35773337	2.092491836	1.96E−11	8.20E−10
rat_circ:chr17:56931036–56944178	*	0.441633884	1.843422885	2.061464294	1.78E−15	1.09E−13
rat_circ:chr8:63507502–63540694	Rec114	0.341030078	1.408824831	2.046521349	8.62E−12	3.74E−10
rat_circ:chr11:59110328–59200801	Lsamp	1.853392371	7.635218572	2.042501119	0	0
rat_circ:chr12:1509719–1512175	Rn5s	3.310525114	13.62707285	2.041343719	0	0
rat_circ:chr2:84361142–84373051	Ankrd33b	0.281535995	1.155090346	2.036614408	3.41E−10	1.28E−08
rat_circ:chr5:39149632–39157890	*	0.252422652	1.033033146	2.032973259	4.32E−09	1.41E−07
rat_circ:chr7:103024404–103123226	*	2.133991155	8.708894234	2.028935355	0	0
rat_circ:chr5:103284367–103352641	Cntln	0.135431589	0.551848363	2.026707619	2.05E−05	0.000354737
rat_circ:chr2:39818337–39845931	*	0.769099545	3.108829149	2.01512909	0	0
rat_circ:chr3:55157605–55224851	*	1.500202608	6.047257552	2.011123669	0	0
rat_circ:chr5:49097739–49187150	*	1.842693006	7.359085633	1.997710785	0	0
rat_circ:chr3:176137456–176143033	*	0.217785593	0.869089421	1.996596103	8.13E−08	2.22E−06
rat_circ:chr5:106553499–106632351	*	1.36441395	5.442020732	1.995861042	0	0
rat_circ:chr16:23448552–23492639	*	1.331199157	5.293003424	1.991360163	0	0
rat_circ:chr4:108913081–108982246	Ctnna2	1.818672104	7.064297787	1.957660702	0	0
rat_circ:chr1:25216049–25284394	Clvs2	1.338072701	5.168828573	1.949680852	0	0
rat_circ:chr8:13085939–13095295	*	0.20772817	0.799366276	1.944159836	7.24E−07	1.68E−05
rat_circ:chr8:110645275–110656857	Ephb1	0.470472499	1.808594153	1.942686405	2.98E−14	1.63E−12
rat_circ:chr7:135919861–135967388	Tmem117	1.354011654	5.136609176	1.923576153	0	0
rat_circ:chr8:130762665–130768360	Snrk	0.921667931	3.483545605	1.918237491	0	0
rat_circ:chr17:29311092–29354416	Fars2	0.834086166	3.147143806	1.915774767	0	0

Following differential expression analysis, 374 DEcircRNAs were identified between the CCI and NC groups (4 mice per group) by using FC filtering (FC ≥ 2 or ≤ 0.5) and FDR (Q < 0.001). The 50 up-regulated DEcircRNAs were listed

**Table 2:** Top 50 upregulated circRNAs between NC and CCI rats

cicName	Gene symbol	NC	CCI	Log2FoldChange	*p* value	*q* value
rat_circ:chr20:37502198–37560959	*	0.25141465	0.003650046	−6.106010103	2.79E−06	5.89E−05
rat_circ:chr7:122834692–122847608	*	0.204582507	0.003650046	−5.808624181	2.64E−05	0.000446745
rat_circ:chr17:50249626–50337400	Sugct	0.182251081	0.003650046	−5.64186876	5.58E−05	0.000866751
rat_circ:chr13:73219583–73265813	Acbd6	0.253446174	0.006087826	−5.379608357	1.32E−06	2.90E−05
rat_circ:chr6:22033342–22038870	Birc6	0.312559274	0.010950139	−4.835108693	8.66E−07	1.97E−05
rat_circ:chr1:215905137–215969554	*	0.350828827	0.012965272	−4.75804289	1.03E−07	2.74E−06
rat_circ:chr17:88963985–88988986	*	4.424488235	0.165307221	−4.742288941	9.11E−79	7.20E−77
rat_circ:chr7:28987514–28992637	*	0.803220892	0.038895817	−4.368109888	6.07E−15	3.56E−13
rat_circ:chr1:127881908–127887913	*	4.246945296	0.216117837	−4.296535473	4.49E−76	3.52E−74
rat_circ:chr1:278580846–278661960	*	1.4349856	0.08427427	−4.089800231	9.09E−25	6.83E−23
rat_circ:chr9:96866448–96884476	Agap1	0.458079355	0.029200371	−3.971538929	3.21E−09	1.08E−07
rat_circ:chr2:62377060–62381205	Golph3	0.251031197	0.018263479	−3.78083314	4.36E−05	0.000704709
rat_circ:chr2:49155620–49170023	*	0.305883775	0.022689226	−3.752904254	1.16E−05	0.000216142
rat_circ:chr13:41898165–41907307	Slc35f5	0.235117703	0.018250232	−3.687396458	1.45E−05	0.00026254
rat_circ:chr17:83502435–83539887	Plxdc2	0.340234629	0.027974186	−3.60436192	3.03E−06	6.33E−05
rat_circ:chr3:94396940–94398038	Hipk3	8.278396821	0.689858769	−3.584978462	3.20E−128	2.59E−126
rat_circ:chr18:32047892–32072938	Arhgap26	0.313075666	0.026103817	−3.584178679	5.92E−06	0.00011622
rat_circ:chr8:23508992–23524947	*	0.274652757	0.029989319	−3.195088096	2.25E−05	0.000387717
rat_circ:chr3:105034310–105085171	*	0.416932296	0.046843127	−3.153903834	7.47E−07	1.72E−05
rat_circ:chr5:58792581–58794404	*	0.523492426	0.060415875	−3.115169064	1.59E−08	4.86E−07
rat_circ:chr5:58467569–58467992	*	1.120383943	0.130229146	−3.104868945	1.07E−16	7.51E−15
rat_circ:chr18:2005294–2047808	Mib1	0.283710612	0.033185498	−3.095795292	4.36E−05	0.000704709
rat_circ:chr9:71954951–71960153	*	0.515480068	0.062050789	−3.054395189	5.79E−08	1.61E−06
rat_circ:chr4:132075419–132113888	Eif4e3	0.350888356	0.044142726	−2.990764449	4.13E−06	8.36E−05
rat_circ:chr2:206534104–206536360	Magi3	0.394038281	0.051861089	−2.925611391	1.41E−06	3.06E−05
rat_circ:chr4:89131241–89138920	Herc3	0.531876619	0.079141743	−2.748580873	8.28E−08	2.25E−06
rat_circ:chr2:207119352–207127766	Slc16a1	0.351063693	0.054750696	−2.680783591	3.12E−05	0.000517084
rat_circ:chr20:14618775–14619985	Gnaz	0.982857873	0.167902134	−2.54936224	8.39E−13	3.99E−11
rat_circ:chr11:89175425–89180448	Spidr	3.926288808	0.687932197	−2.512828014	4.79E−44	3.71E−42
rat_circ:chr17:34787652–34799549	Exoc2	0.432093695	0.075805009	−2.510979092	9.81E−06	0.000186663
rat_circ:chr6:57016029–57048318	Agmo	0.595587198	0.107379529	−2.471593757	1.66E−07	4.32E−06
rat_circ:chr18:79813606–79815529	Zfp516	0.60811866	0.113151438	−2.426097934	5.17E−08	1.44E−06
rat_circ:chr3:101926186–101954565	*	0.39540261	0.075194562	−2.394622161	5.47E−05	0.000857167
rat_circ:chr10:6991635–6992636	Usp7	0.360561779	0.068704837	−2.391762898	3.19E−05	0.000527475
rat_circ:chr6:30213961–30219724	*	0.39272531	0.075423859	−2.380427707	5.47E−05	0.000857167
rat_circ:chr4:2546232–2564215	*	0.396666041	0.083245965	−2.252472648	3.10E−05	0.00051578
rat_circ:chr14:85057352–85061960	Nf2	2.033181317	0.433870486	−2.228402524	6.13E−21	4.47E−19
rat_circ:chr14:39730079–39755962	Gabra2	0.646023427	0.138812971	−2.218444102	1.58E−07	4.11E−06
rat_circ:chr9:92380210–92405119	Trip12	0.399681023	0.086691517	−2.204886337	5.47E−05	0.000857167
rat_circ:chr11:24479964–24498116	App	1.540401399	0.348960903	−2.142169025	4.12E−15	2.49E−13
rat_circ:chr10:72203091–72212356	Myo19	0.880382639	0.205470222	−2.099201373	1.80E−08	5.47E−07
rat_circ:chr1:220039308–220042767	Rbm4	0.760440921	0.182218718	−2.061165006	1.10E−07	2.94E−06
rat_circ:chr18:24611733–24614740	Wdr33	0.454872734	0.11004188	−2.047410267	1.53E−05	0.000274117
rat_circ:chr10:94758719–94760369	*	0.708886324	0.188218263	−1.913147675	8.76E−07	1.99E−05
rat_circ:chr20:50725400–50764570	Hace1	0.639217189	0.171416942	−1.898796495	4.67E−06	9.39E−05
rat_circ:chr3:157028112–157040380	Chd6	0.521580243	0.142758265	−1.869314953	1.21E−05	0.000224614
rat_circ:chr18:13352206–13417002	*	0.85873703	0.23861058	−1.847558389	1.72E−07	4.43E−06
rat_circ:chrX:159765411–159801803	Arhgef6	0.560013063	0.161390973	−1.794900589	4.07E−05	0.000661291
rat_circ:chr19:25886356–25886887	Nfix	16.23637991	4.685799745	−1.792862798	3.39E−116	2.71E−114
rat_circ:chr16:2158111–2179241	Slmap	0.859120326	0.269488853	−1.672634608	5.16E−07	1.22E−05

Following differential expression analysis, 374 DEcircRNAs were identified between the CCI and NC groups (4 mice per group) by using FC filtering (FC ≥ 2 or ≤ 0.5) and FDR (Q < 0.001). The 50 down-regulated DEcircRNAs were listed

### Functional annotation of DEcircRNAs

**Fig. 4:**
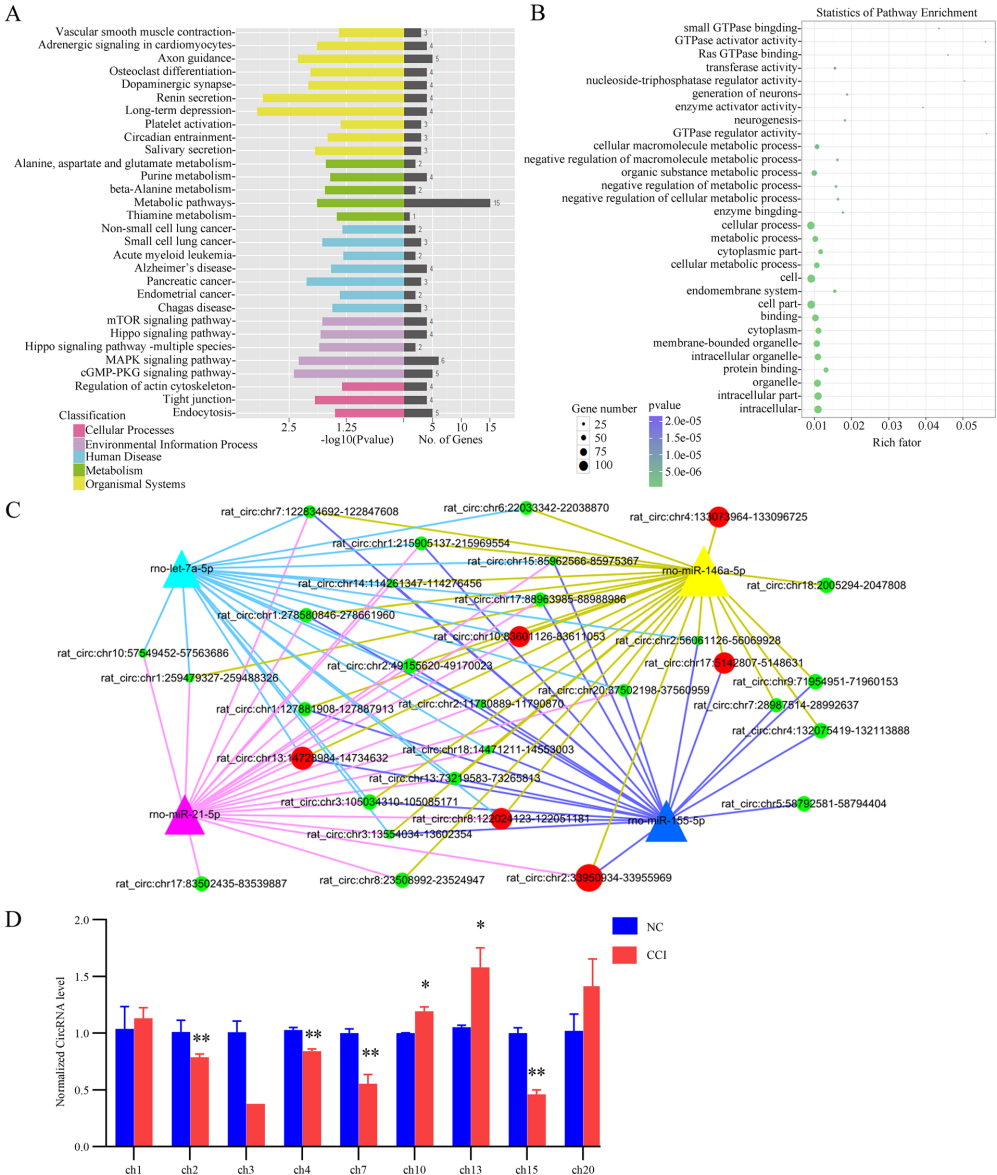
Differentially expressed circRNA functional analysis between NC and CCI rats. **a** Kyoto Encyclopedia of Genes and Genomes (KEGG) pathways enriched by DEcircRNAs. The vertical axis is the pathway category. The horizontal axis is the −log10 (*p* value) of the pathway and the number of differentially expressed circRNAs (DEcircRNAs). Different colors are used to distinguish cellular processes, environmental information processing, human diseases, metabolism and organismal systems. **b** The most enriched Gene Ontology (GO) terms of DEcircRNAs. The vertical coordinate is the enriched GO terms, and the horizontal coordinate is the rich factor. Rich factor value represents the enrichment degree. The size of the points represents the number of DEcircRNAs in the term. The colors of the points correspond to different *p* value ranges. **c** CircRNA–microRNA competing endogenous RNA network. The red nodes represent the upregulated circRNAs, and the green nodes represent the downregulated circRNAs. The size of each node represents the functional connectivity of each circRNA. The line color indicates potential interactions between circRNAs and microRNAs, which were predicted by using TargetScan and miRanda. **d** CircRNA expression changes validated by quantitative PCR (qPCR). qPCR analysis of differences in the expression levels of DEcircRNAs in the dorsal root ganglion between the CCI model and NC groups. The results were calculated by normalizing to GAPDH in the same sample with the 2^−ΔΔCt^ method. Changes in relative levels of circRNAs expressed as fold of the controls. All values are expressed as the mean ± SEM, Student’s *t* test, *p < 0.05 (*n* = 3)

To predict the functions and pathways related to the DEcircRNAs identified above, KEGG and GO analyses were performed for the host genes of DEcircRNAs. According to our results, the most significantly enriched KEGG pathways included ‘dopaminergic synapse,’ ‘renin secretion,’ ‘long-term depression,’ ‘metabolic pathways,’ ‘mitogen-activated protein kinase (MAPK) signaling pathway,’ and ‘cyclic guanosine monophosphate-protein kinase G signaling pathway.’ Next, we performed GO analysis of the linear counterparts that produced DEcircRNAs. Our data showed that the most significantly enriched GO terms included ‘generation of neurons,’ ‘neurogenesis,’ ‘GTPase regulator activity,’ ‘negative regulation of metabolic process,’ and ‘cellular process.’ The top 30 significantly enriched KEGG and GO terms are shown in Fig. 4a, b. The results suggest that these signaling pathway and molecular function-related to DEcircRNAs may be involved in the pathogenesis and development of chronic pain.

### CircRNA–miRNA competing endogenous RNA network

There is emerging evidence that miRNAs play an important role in the development of chronic pain. In our previous study, we found that miRNA-146a-5p, a key molecule in the negative regulation of innate immunity, has dysregulated expression in the DRG of rats with chronic pain [32]. In addition, the aberrant expression of other miRNAs in DRG has also been reported, such as miR-155-5p [33], miR-21 [34], and let-7a-5p [35]. Several circRNAs have been recently identified that exert important regulatory effects as miRNA sponges. Potential connections between circRNAs and microRNAs involved in chronic pain were predicted by using TargetScan and miRanda, and the interaction results were displayed by Cytoscape. Four chronic pain-related miRNAs exhibited an interaction with the top 50 circRNAs according to the size of *p* value (Fig. 4c and Additional file 4: Table S4).

### Validation of circRNA expression using qPCR

To validate the screened circRNA candidates, qPCR analysis was performed to amplify the back-spliced junctions of nine typical DEcircRNAs (chr1:166208849–166259335, chr2:26590643–26650618, chr3:112752502–112762808, chr4:132075419–132113888, chr7:64125692–64131186, chr10:8331904–8371302, chr13:84161876–84168811, chr15:51936366–51944899, and chr20:50725400–50764570). As detected by circRNA HTS, these circRNAs were expressed in all CCI and NC samples and showed significant expression differences between groups. In addition, all of these circRNAs were predicted to have at least one binding site with miR-146a-5p. According to our qPCR results, with the exception of chr10:8331904–8371302, chr13:84161876–84168811, and chr20:50725400–50764570, the expression levels of all remaining DEcircRNAs were consistent with those measured by sequencing analysis (Fig. 4d).

## Discussion

CircRNAs are mainly alternative splice variants of pre-mRNA [36]. Alterations in universal circRNA expression have been identified in various diseases, including cancer [37], Alzheimer’s disease [38], and type 2 diabetes [39]. In our study, we revealed circRNA expression changes at the genome-wide level by comparing circRNA expression in the DRGs of CCI and NC rats.

The CCI is one of the most frequently used models of neuropathic pain [40]. As shown by our pain behavior test, PWMT and PWTL were significantly decreased in the CCI group compared with the NC group, indicating that the CCI rat model was successfully established. CircRNA samples were prepared from the DRGs of CCI and NC rats and were subjected to circRNA sequencing. Differential expression analysis identified upregulated expression changes in 290 circRNAs and downregulated expression changes in 84 circRNAs of DRG neurons of CCI induced neuropathic pain rats compared with the control group. The number of downregulated DEcircRNAs was great less than that of the upregulated ones. We listed top 50 upregulated DEcircRNAs and top 50 downregulated DEcircRNAs respectively. Among these DEcircRNAs, circ:chr13:14728984–14734632 in CCI rats was upregulated more than tenfold and the circ:chr20:37502198–37560959 in DRG of CCI rats was downregulated more than 12 fold compared with the rats in control group. In order to confirm the reliability of our sequencing data, we picked nine circRNAs with high expression level and significant difference in the expression between CCI and NC groups. Expression patterns of these circRNAs was performed by qPCR and the consistent trends were compared between qPCR results and the circRNAs HTSeq.

As we known, circRNAs could expressed dynamically and exert their function through multiple mechanisms [41]. However, the vast majority of circRNAs are generated from the sequences of their host genes, and some circRNAs also play roles in transcriptional or posttranscriptional regulation of their host genes [36]. Therefore, the functions of circRNAs may be relevant to their host genes. In our study, we first conducted the GO analysis and KEGG pathway analysis for the host genes of the DEcircRNAs to predict the function of them. According to our results, a wide variety of KEGG and GO terms were identified, indicating a broad range of functional modulations in the DRG of neuropathic pain rats. The GO analysis indicated that the DEcircRNAs were presumably involved in development of neurons and nervous tissue, the regulation of GTPase, metabolic and cellular process.

The top five significant functional terms conducted by KEGG pathway annotation included ‘dopaminergic synapse,’ ‘renin secretion,’ ‘MAPK signaling pathway,’ and ‘neurogenesis.’ It could be reasonable, as many of these processes were considered contributing to the development and maintenance of chronic neuropathic pain. Dopaminergic functions are commonly modulated in neuropathic pain, and administration of dopaminergic agents may relieve neuropathic pain [42]. Renin is a component of the renin–angiotensin–aldosterone system, and increased levels of renin are associated with neuropathic pain [43]. Increased phosphorylation of MAPK in the DRG is an early event in neuropathic pain following peripheral nerve injury, and the extracellular signal-related kinase/MAPK pathway is considered a novel therapeutic target for neuropathic pain [44]. Dysregulation of neurogenesis in the hippocampus is also suggested to be involved in neuropathic pain [45]. Therefore, we hypothesized that the biological functions of DRG are widely modulated in neuropathic pain.

In our and others’ previous research, miRNAs were proved to play an important role in development of neuropathic pain. miR-146a and miR-155, taking a crucial part in inflammation, immunity as well as tumor, were highly expressed in DRG neurons of chronic neuropathic pain rats [32, 33]. miR-21 may serve as an endogenous ligand, acting on TLR8 in endosomes and lysosomes, and involve in the maintenance of neuropathic pain [34]. Recent evidence revealed that the essential function of circRNAs is serving as miRNAs sponges. We predicted the circRNAs/miRNA interaction using TargetScan and miRanda and then constructed ceRNA network. According to our results, we deduced that these circRNAs may bind pain related miRNAs and inhibit their biological functions. Our results implied that it is essential and worthwhile to further study potential functions of these pain related DEcircRNAs in chronic neuropathic pain. Gao et al. analyzed the circRNA expression in the dorsal horn of the spinal cord using microarrays. They only used fold changes ≥ 2 as selection criterion to select significant DEcircRNAs and found expression change of 469 circRNAs in CCI rats compared with sham-operated rats. Similar to our KEGG pathway annotation results, they also reported the MAPK signaling pathway was related to neuropathic pain [14]. However, we first identified circular RNA expression profiles of the dorsal root ganglion in a rat model of neuropathic pain. Our results may help to elucidate peripheral mechanism of neuropathic pain.

The main strength of this study was that circRNA expression in DRGs was compared between CCI and NC rats, which provided a global picture of circRNA expression alterations in DRG. The limitation of our study was that the CCI model was the only neuropathic model included in our study. Thus, additional neuropathic models are required in future studies to validate our results. Further functional studies in vivo should also be performed to elucidate the roles of DEcircRNAs in neuropathic pain.

## Conclusions

We developed a CCI model and identified 374 significant DEcircRNAs in the DRGs of CCI and NC rats. These DEcircRNAs may be involved in various biological pathways and functions, which are potentially modulated in DRGs by neuropathic pain. Further bioinformatics analyses suggested that these DEcircRNAs could be sponged with miRNAs, which targeted mRNAs associated with chronic pain. Our study may provide new insights into the molecular mechanisms underlying neuropathic pain.

## Supplementary Information

**Additional file 1: Table S1.** List of DNA primer sequences designed for qPCR.

**Additional file 2: Table S2.** List of total circRNAs detected in the DRG of NC and CCI rats. RNA-seq was performed to profile circRNA expression in four paired DRG samples. A total of 13,172 circRNAs were detected in the L4–L5 DRG samples from the CCI and NC groups (4 mice per group).

**Additional file 3: Table S3.** List of DEcircRNAs were identified between the CCI and NC groups. Following differential expression analysis, 374 DEcircRNAs were identified between the CCI and NC groups (4 mice per group) by using FC filtering (FC ≥ 2 or ≤ 0.5) and FDR (Q <  0.001).

**Additional file 4: Table S4.** List of circRNAs and miRNA predicted by using miRanda and TargetScan. Potential connections between circRNAs and microRNAs were predicted by using TargetScan and miRanda, and the interaction results were displayed by Cytoscape. All the circRNAs and miRNAs were listed.

## Data Availability

There are no data, software, databases, or applications/tools available apart from those reported in the present study. All data are provided in the manuscript and supplementary data section.

## Abbreviations

cGMP: Cyclic guanosine monophosphate
ERK: Extracellular signal-regulated protein kinase
MAPK: Mitogen-activated protein kinase
PKG: Protein kinase G
